# High density and successful breeding of Turtle doves *Streptopelia turtur* in Moroccan olive groves

**DOI:** 10.7717/peerj.14375

**Published:** 2022-11-11

**Authors:** Wafae Squalli, Michael Wink, Ismail Mansouri, Fatima Fadil, Mohamed Dakki

**Affiliations:** 1 Laboratory of Functional Ecology and Environmental Engineering, Department of Biology, Faculty of Sciences and Techniques Fez, Université Sidi Mohamed Ben Abdellah, Fez, Morocco; 2 Institute of Pharmacy and Molecular Biotechnology, Heidelberg University, Heidelberg, Germany, Germany, Germany; 3 Study Centre of Bird Migration, Geo-Biodiversity and Natural Patrimony Laboratory, Department of Zoology and Animal Ecology, Scientific Institute, Mohammed V University in Rabat, Morocco, Rabat, Morocco

**Keywords:** Population, *Streptopelia turtur*, Breeding, Olive groves

## Abstract

**Background:**

The turtle dove is a migratory species that has suffered a rapid decline principally across its Northern ranges, despite pronounced conservation measures. Consequently, it has been categorized as ‘Near Threatened’ in Europe. Degradation of breeding habitats and a decrease in food resources are listed as principal causes of this decline. Despite its importance, the productivity of the North African population is widely unknown. Here we present the first estimation of the density of the breeding population and the superior reproductively of *Streptopelia turtur arenicola* in Morocco and entire North Africa.

**Methods:**

This study was carried out for two seasons 2018–2019 in the Saïss plain, central Morocco. Based on previous data, doves were monitored weekly, from early March to late August, in aquatic ecosystems (two dams and one river) and farmlands (cereals and orchards). The breeding population was censused using the “point-count” method, following a walked transect of 5 km in orchards, 7 km in cereal fields, and 3 km along the river. Equally, nests were searched in natural habitats counting riparian trees, forests, and ornamental trees, and in orchards based on the Common Birds Census (CBC) methodology, in which the singing doves, mating pairs, nesting, and/or feeding behavior were the most monitored signs to discover nests. In orchards, nests were searched line-by-line based on the rows of fruit trees. For each recorded nest, we note the breeding chronology, clutch size and incubation period, success and failure factors, dimensions, and vertical placement on trees. To evaluate the predictors of doves’ occurrence, we noted at each site the presence of cereals, water, human disturbance, presence of nesting trees, and predators.

**Results and Discussion:**

In total, 3,580 turtle doves (22.37 birds/ha), including 240 breeding pairs, were documented. Nesting occurred mainly in olive groves, cereals were used for forage, and aquatic ecosystems for water sources. The nesting period lasted from late April to July (last fledglings). All nests were located on olive trees at a height of 225.30 ± 48.87 cm. The clutch size was 1.98 ± 0.13 (laid eggs/built nests), the incubation period lasted 14.16 ± 1.32 days, and the rearing period lasted 16.54 ± 1.76 days. The breeding success among the 240 monitored nests accounted for 73.84% during the nesting phase and 87.42% during the incubation phase; 71.5%% of nestlings have fledged, which is the highest success rate for turtle doves in Europe and Northwest Africa. Clutches were aborted mostly due to predation from snakes (7.5% of nests, 16.12% of eggs, and 5.63% of chicks), nest desertion (9.16% of nests and 5.37% of eggs), and marginally by the destruction of nests through farming activities. These findings are important for conservation plans, to restore turtle doves’ habitats in Europe, where the species is widely declining.

## Introduction

The turtle dove *Streptopelia turtur* breeds in Europe (Browne & Aebischer, 2004; Schumm et al., 2021), western and central Asia (Abode et al., 2021) and northern Africa (Squalli et al., 2022). It winters in Africa mainly in the Sahel zone with new attempts recorded currently in North Africa (Schumm et al., 2021; Mansouri et al., 2022a). Four subspecies are recognized: *S. t. turtur* (Europe, S and N Mediterranean coast, Asia Minor to W Siberia) (del Hoyo et al., 2014; Marx, Korner-Nievergelt & Quillfeldt, 2016); *S. t. arenicola* (Morocco to Tripolis) (El Hassani, Dakki & El Ghadraoui, 2018); from Iraq, Iran, Afghanistan to W China (Abode et al., 2021); *S. t. hoggara* (Ahaggar, Tibesti, Ennedi Massifs in Sahara (Chedad, Bendjoudi & Guezoul, 2020) and *S. t. rufescens* (Kufra Oasis, Dakla, Kharga and Faiyum oases, Nile valley) (Dubois, 2002; Prakas et al., 2021).

The turtle dove is a typical migratory species that has suffered a rapid and continuous decline principally across its Northern ranges (population losses: −78% in Britain from 1980 to 2013 (Browne & Aebischer, 2005) and −70% in Spain from 1980 to 2020; Moreno Zarate et al., 2020; Moreno Zarate, Arroyo & Peach, 2021), despite pronounced conservation measures (Hanane, 2017; Moreno Zarate, Arroyo & Peach, 2021). Consequently, turtle doves have been categorized as ‘Vulnerable’ in Europe (BirdLife International, 2015, 2019). Degradation and loss of breeding habitats (Browne et al., 2004), decrease of food resources (Browne & Aebischer, 2003; Eraud et al., 2009), intensification of agricultural activities (Mansouri et al., 2020b), and unsustainable hunting (Boutin & Lutz, 2007; Moreno Zarate, Arroyo & Peach, 2021) are listed as principal causes responsible for this decline. Some of the factors are relevant both in the breeding habitats as in the wintering areas in Africa (Dunn & Morris, 2012; De Vries et al., 2022).

The breeding biology of turtle doves has been widely studied in Europe and Northwest Africa (Gruychev & Mihaylov, 2019; Dunn et al., 2021; Squalli et al., 2021; Mansouri et al., 2022a; Squalli et al., 2022). Breeding turtle doves were monitored in different ecosystems, including farmlands (Mansouri et al., 2019, 2020b, 2021b), riparian systems (Chiatante, Porro & Meriggi, 2021; Mansouri et al., 2021b; Squalli et al., 2022), and forests (Moreno Zarate et al., 2020; Tellería et al., 2020; Mansouri et al., 2021b). Generally, breeding success was low (Hanane, 2017; Mansouri et al., 2021b). This reduced reproductive success is also considered as the main factor limiting the recovery of the species (Hanane, 2017). However, with the continuous decline of turtle doves, mainly in Europe, a detailed analysis of their breeding biology and ecology and failure factors are urgently needed. Changes of habitat structure (Dunn & Morris, 2012), climate conditions (Eraud et al., 2009), and agricultural practices (Mansouri et al., 2021b), both in the breeding areas and in the wintering regions appear to be responsible for the decline of turtle doves and need more attention.

In this communication we report the results of our field study of the North African subspecies of turtle dove *Streptopelia turtur arenicola* in farmland and natural habitats surrounding the prehistoric city Fez in central Morocco. The breeding bioecology, occurrence, and density of turtle doves were assessed in this peri-urban rea, rich in cereal fields, aquatic habitats, and orchards. We aimed to investigate in detail the habitat use, breeding performances, and governing factors.

## Materials and Methods

### Study area

**Figure 1:**
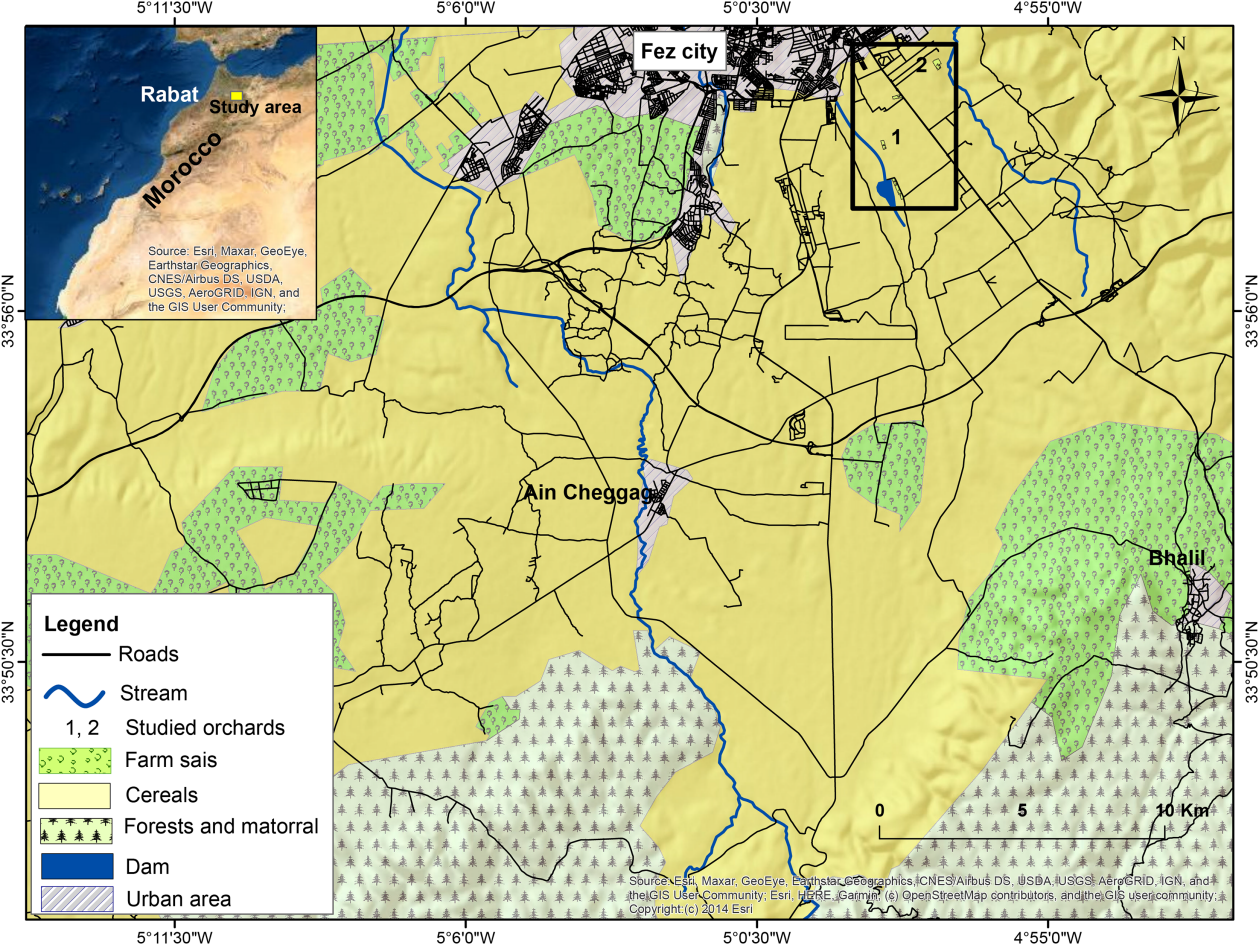
Map showing the location of the Saïss plain in the vicinity of Fez (Morocco). Map source credit: Esri, Maxar, GeoEye, Earthstar Geographics, CNES/Airbus DS, USDA, USGS, AeroGRID, IGN, and the GIS User Community; Esri, HERE, Garmin, ©OpenStreetMap contributors, and the GIS user community; Copyright:© 2014 Esri.

The fieldwork was carried out in an irrigated area (117.500 ha) of the Saïss agricultural plain (Fig. 1) situated in the periphery of the historical city Fez in Central Morocco (Kessabi et al., 2022). The region is at a 600 m altitude above sea level and is located between the Rif’s and Middle Atlas Mountains (Dauteuil, Moreau & Qarqori, 2016). The selected zone is rich in wetlands, including RAMSAR sites (Middle Atlas lakes) and sites of biological interest (Mahraz dam, El Gaada dam, and Allal Fassi Dam, etc.) (Cherkaoui et al., 2014; Squalli et al., 2021).

The Saïss plain has a semi-arid climate. Temperatures vary widely, from moderate temperatures during winter to up to 45 °C in hot summers. Cereal crops dominate the plain and are used for food consumption and industry (56.940 and 31.423 ha). Wheat (*Triticum turgidum* and *T. aestivum*; 45.075 ha), fodder crops of alfalfa (*Medicago sativa*), and maize (*Zea mays*; 23.000 ha) are the primary crops in the area (Kouchou et al., 2020; Hossard et al., 2021). Olive groves (*Olea europea*) occupy 11.574 ha (Daoui & Fatemi, 2014). In this agricultural plain, olive orchards are distributed in patches (belonging to a farm or a group of farms located near each other) throughout the region.

For our study of the breeding biology of turtle doves, two study orchards (8,901 m^2^) were selected and monitored from 2018 to 2019; more study sites were not included due to the absence of nests in other habitats. Within olive groves we distinguished two areas: the boundary zone including the first three olive rows on each side of the orchard, and the central zone including the internal rows of the olive trees.

### Data collection

Based on previous data on the breeding chronology of turtle doves in Morocco (El Hassani, Dakki & El Ghadraoui, 2018), we searched for nests from early March to late August (corresponding to breeding season of turtle doves in Morocco and Northwest Africa; Mansouri et al., 2020a, 2020b) between 2018 and 2019 and recorded presence of singing doves, mating pairs, nest building, provisioning of food for chicks, and followed the recommendation of the Common Birds Census (CBC) methodology (Calladine, Buner & Aebischer, 2010; Mansouri et al., 2020b, 2021b). In our case, we based our assessment on both direct observations and acoustic calls.

**Figure 2:**
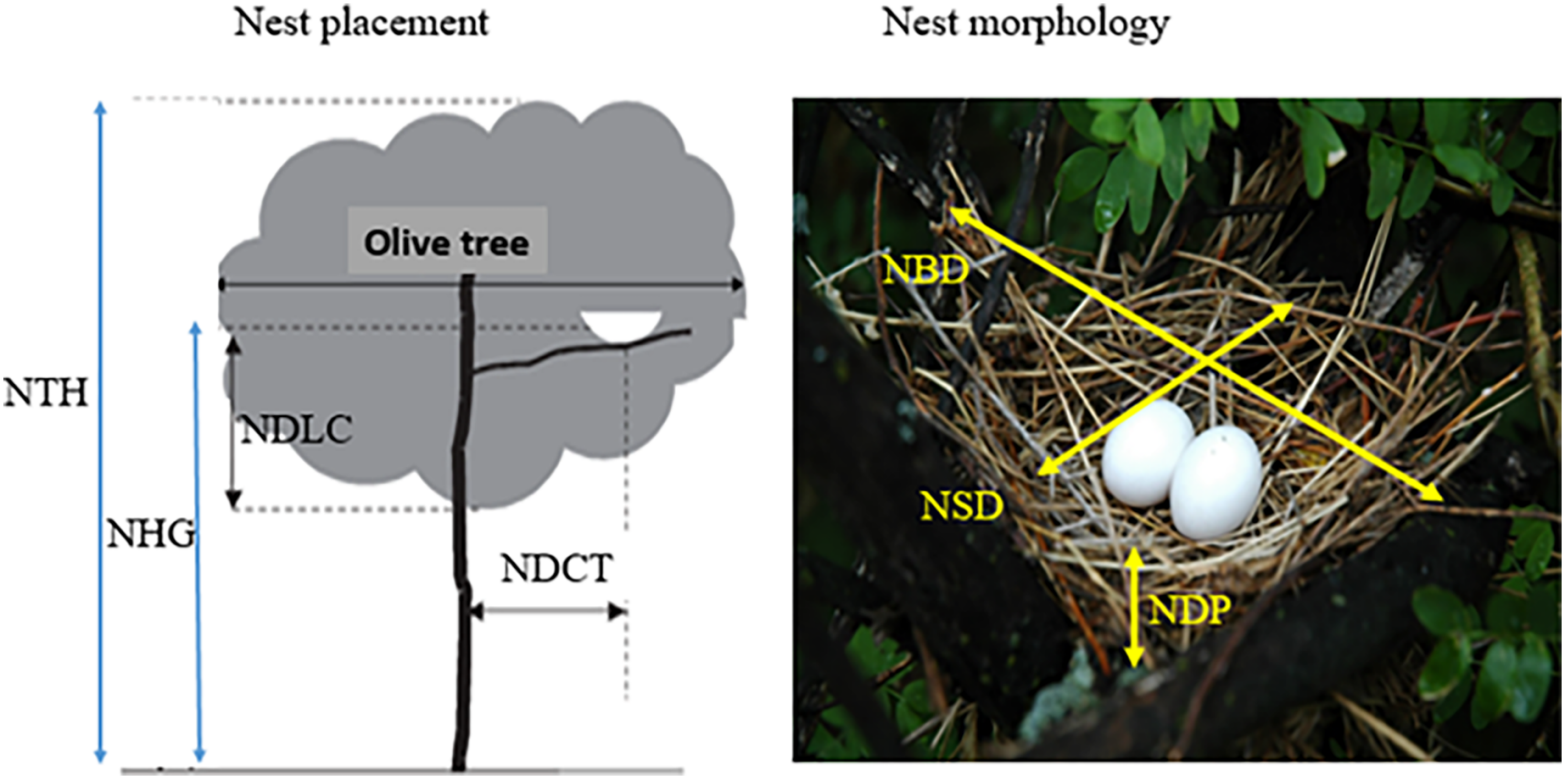
Nest placement parameters. Nesting-tree height (NTH), nest height above ground (NHG), distance to lower canopy (NDLC), distance of nest to olive central trunk (NDCT) and nest dimensions (big diameter (axis) (NBD), small diameter (axis) (NSD) and depth (NDP)).

For nesting parameters, during every single visit (one visit per week for each orchard), we recorded nest placement (Fig. 2) (five parameters: nesting-tree height (NTH), nest height above the ground (NHG), lower canopy distance (NDLC), and nest distance to central trunk (NDCT)), nest size (three parameters: nest axis (big and small diameters) and nest cup depth). For breeding phenology, we recorded three parameters: dates of nest construction, egg laying, and fledging of chicks. For nest status, we recorded three parameters: (new-empty nest, nest with eggs or chicks), and for breeding success, we recorded failure causes: (predation, abandonment, destruction, and death of clutch). The documentation of failure factors, was based on direct and indirect observations of egg’ shells, ruminants of feathers (birds of prey don’t eat feathers of small birds) and exoskeletons (for reptilians and mammalians who don’t eat exoskeleton; Dondini & Vergari, 2005), or human paths around nesting sites. Then, the breeding success was determined for the different phases of the breeding cycle: nest construction (occupied nests/total built nests), egg laying (hatched eggs/total laid eggs), and fledging (fledged broods/total hatched chicks). Furthermore, nest sizes (Fig. 2) were measured when breeding birds were absent from the nests (when the breeding pairs departed for food research principally during morning). In addition, farming practices inside orchards (tree cutting, harvesting, pesticide use, grazing, *etc*.) were documented during the breeding periods. Equally, we monitored if disturbed nests were deserted or not (two visits after last disturbance by farmers and if the nest was deserted the cause was mentioned).

To evaluate the population size and population density and habitat use in the Saïss plain (nearly 70 km^2^), the study area was divided into three principal habitats, including aquatic ecosystems (two dams, river, and other streams), farmlands (cereals and orchards) and urban systems (Fig. 1). In each site, we censused turtle doves regularly using the “point-count” method (10–20 min): We walked a transect of 5 km in orchards, 7 km in cereal fields, and 3 km along the river (the distance of each transect was chosen based on the structure of each habitat and its importance in the area). Transects were long in cereals because of their extensive surfaces and open terrain in the **Saïss** plain, 5 km of transect was used in grouped orchards (isolated orchards of small surfaces were sampled with simple visits), and short transects were used in aquatic ecosystems due to their limited surfaces (*i.e.*, Fez river is only 9 km of length). Dams were monitored by fixed observational sites due to the small size of these habitats.

In total, 64 transects for each habitat and a total of 740 “point-counts” (a medium of four points per transect separated by 0.7 to 1.2 km) were realised during breeding seasons of 2018 and 2019. After the observation of birds in each point, the distance between adjacent points was recorded too and the number of doves were considered for mixed habitats (*i.e.*, each individual found between two different habitats was assigned for mixed habitat that combining between the adjacent habitats). The observation points of isolated orchards were added to those of transects in grouped orchards (similar habitats), while “point-counts” of dams were separated from those of rivers’ transects (considered as different habitats despite their richness in water). This mixture of methods and the high number of observations aimed to census the turtle doves as completely as possible (Bani et al., 2006) and to avoid a double counting of birds. In addition, we were able to collect a wide range of other ecological data in a cost-effective manner (Mansouri et al., 2021a). Each particular transect walk lasted about 8 h (from 06:00 to 18:00 h (with a break between 11:00 and 15:00 h, because turtle doves are less active during this period)) (Mansouri et al., 2019). As mentioned before we recorded the numbers of doves seen and/or heard singing, behaviour (nesting, feeding or courtship to document habitat use), distance to cereals and water sources (foraging resources for doves; Mansouri et al., 2019), occurrence of predators (we noted (observed or heard) aerial or terrestrial predators on the same observation point of doves), occurrence of competitors (CP) (other Columbidae that have similar ecological requirements; Mansouri et al., 2022a), distance to nearest road (DR: National, regional, or local roads) and nearest building (DB: depots, homes, and all kind of human buildings), and potential nesting trees (necessary element for breeding). These ecological factors are assumed to explain the habitat use in turtle doves as mentioned currently in Morocco (Mansouri et al., 2022b).

To determine distances from observation points to ecological factors, transects were recorded in mobile phone Geotraker software. Further, the Geotraker circuits, including picked observation points, were reported in Google Earth Pro. Then, we used zoom button to approach the observation point and we calculated distances with path (we measured distances between fixed observation point and potential factor with help of drawn path on the maps extracted from satellite imagery of Maxar (open sources)). In the same way, we calculated the surfaces of sampled habitats *via* polygons covering each habitat on the same satellite images (used later to calculate the densities of doves per ha).

### Statistics

We calculated the densities as the number of observed doves per ha of studied habitat (number of observed or heard doves per transect/ha of habitat type), while density of breeding doves was only calculated for olive orchards were nesting activity was observed (number of breeding pairs/ha of olives) during 2018–2019 breeding seasons. Breeding pairs were extracted from number of detected nests. In addition, we calculated the occurrence probability for principal habitats (cereals, olives, and aquatic ecosystems) and their buffer zones (intermediate zones between two adjacent habitats). We tested for normality and homogeneity of variance for all examined variables *via* Kolmogorov–Smirnov test. For all these variables, we considered two nesting orchards and two breeding seasons (we could not record any effect of the orchard (two olive orchards) or season (2018–2019) on breeding performances). The one way ANOVA test was used to assess differences in success rates among nesting (active nests/built nests), laying (hatched eggs/laid eggs), and fledging (chicks leaving their nests/fledged chicks) phases. Similarly, failure factors were analyzed by ANOVA among the three breeding phases. The multiple range test was used to compare the occurrence probability of turtle doves between monitored habitats. Correlations between nest placement parameters and nest morphology were analyzed *via* Pearson correlation coefficients (*n* = 120). Relationships between reproductive success (total-fledged chicks/total laid eggs per nest) and failure variables (predation attacks (quantitative; the number of attacked nests, eggs and chicks) and nest desertion (qualitative; abandoned/ built nests) were involved due to their significance in terms of failed proportions of clutches) were assessed using a generalized linear model (GLM). Furthermore, a general linear model was used to test the relation between laying and fledging to nesting dates. Finally, to examine the potential factors capable of influencing breeding or occurring doves in the Saïss plain, generalized linear models were applied. The cereals (presence (1) or absence (0)), potential nesting trees (planted or natural) (presence (1) or absence (0)), predation attacks (attacks (1) or not (0)), availability of water (presence (1) or absence (0)), and human presence in the habitat (infrastructure or human activity near the area (1 or 0)) were tested (response variables: 0 (no bird observed in the habitat) and 1 (at least one dove is observed)). Poisson distribution error (sum data) was used with identity link and interactions between different potential predictors. Prior modeling, collinearity between predictor variables was tested using a variance inflation factor (VIF) that quantifies the extent of increase in the variance arising out of collinearity of multiple explanatory variables (James et al., 2013). Variables with a VIF ≥ 10 were not accepted (Hair et al., 1995). To determine the optimum breeding activities in turtle doves, nesting (*n* = 120 nests), laying (*n* = 186 eggs), and fledging (*n* = 142 chicks) activities were considered as dependent variables, while breeding dates (*n* = 17 weeks between March and August) were considered as independent variables, and were tested with principal component analysis (PCA). Only components with eigenvalue >1 were considered. To identify the spatial limits of micro-nesting-niche of turtle doves, we considered nests (120) as dependent variable and we divided them following orchards (N1 = 58, N2 = 62 nests) and breeding season (2018 = 55 nests, 2018 = 65 nests), while nesting parameters (nest big diameter (NBD), nest depth (ND), nest height (NH), nesting tree height (NTH), nest small diameter (NSD), and lower canopy distance (NDLC)) were considered as determinants (factors) of nesting niche, then the detrended correspondence analysis (DCA) was used to compare the variation of nest-niche limits (Antonini & Martins, 2003; Hanane & Yassin, 2017). In DCA, we selected 2D plots and we selected the convex hull to distinguish between orchards (N1 and N2) and seasons (2018 and 2019). Tests were done in STATGRAPHICS Centurion software, version XVI. I and results were given as sample size and mean ± SD.

## Results

### Breeding population and habitat use

**Figure 3:**
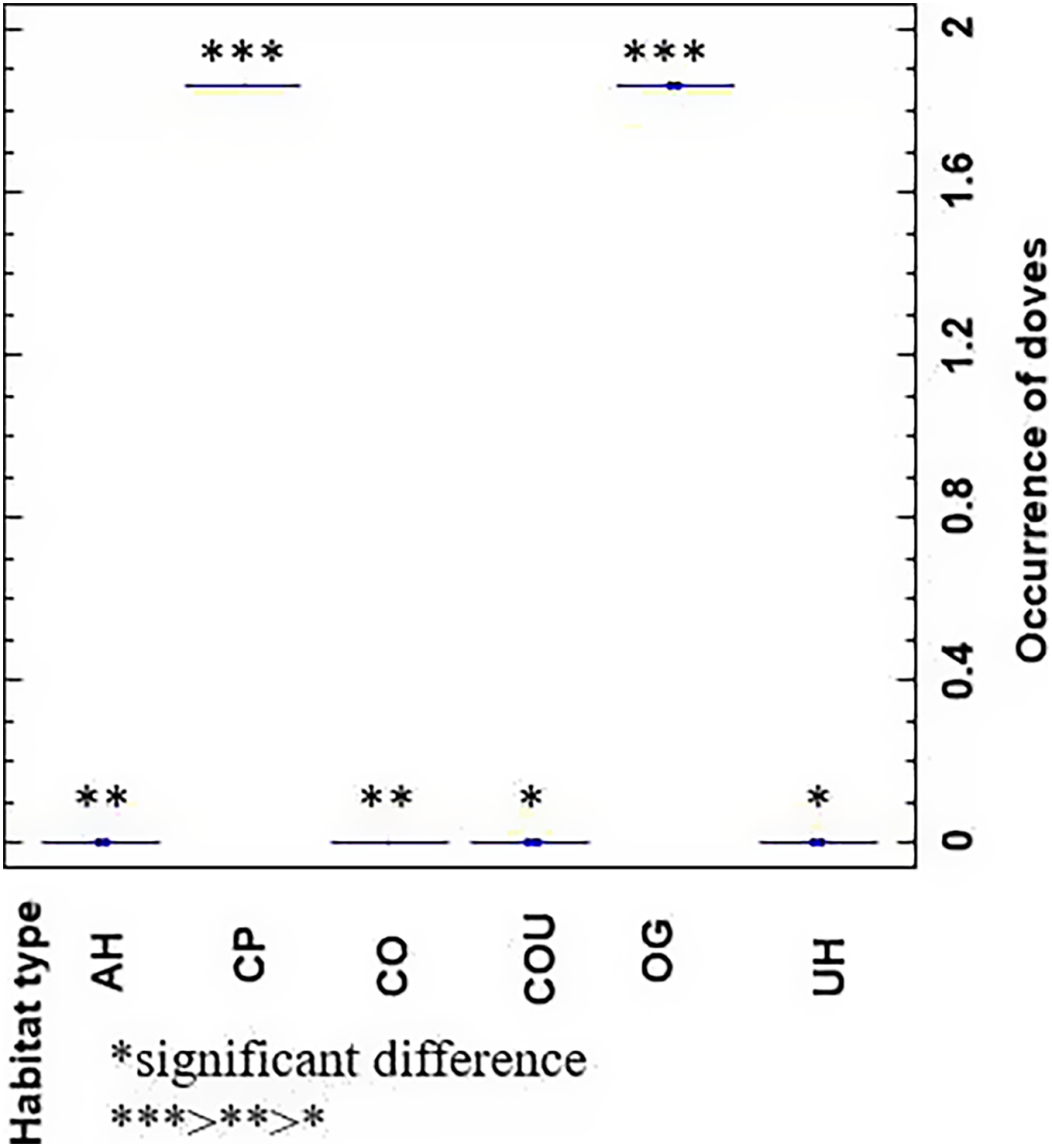
Comparison of occurrence probability of turtle doves in studied habitats. AH, aquatic or riparian habitats; CP, cereal plots; CO, cereal-olives; COU, cereals-olives-urban; OG, olive groves; UH, urban habitat.

Across two seasons, 3,580 turtle doves were observed, and 240 breeding pairs were confirmed (120 nests were documented for both orchards). In detail, 116 (58 nests) and 124 pairs (62 nests) were documented in orchards 1 and 2, respectively, with 55 nests in 2018 and 65 nests in 2019. The higher densities of turtle doves were recorded in cereal farms and olive orchards (Table 1). A small number of turtle doves was documented near rivers and dams. The occurrence of turtle doves is higher in cereal plots (CP) and olive groves (OG), followed by aquatic ecosystems (AH) and habitats with mixed cereals and olives (CO) with moderate occurrence rates (Fig. 3). In contrast, a lower occurrence was recorded in urban habitats and mixed systems combining cereals, olives, and urban areas.

**Table 1:** Population density of turtle doves at the Saïss plain and selected habitats between 2018 and 2019.

Parameters	Habitat surface (ha)	Observed turtle doves	Observed doves/ha	Breeding doves/ha	Activity of doves in each habitat
**Olives**	22	500–550	23.86	0.026	Nesting-feeding-resting
**Rivers**	14	260–300	20	–	Feeding-nesting
**Dams**	8	140–180	20	–	Feeding-nesting
**Cereals**	24	800–850	34.375	–	Feeding

**Figure 4:**
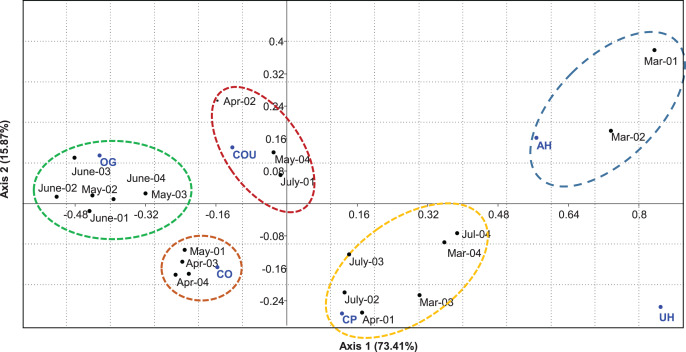
Spatio-temporal use of habitats by breeding doves in the Saïss plain. OG, olive groves; CO, cereals and olives; COU, cereals-olives-urban; CP, cereal plots; AH, aquatic ecosystems; UH, urban habitats.

After their arrival in March, migrant turtle doves occupied riparian areas of aquatic ecosystems (AH). They displaced into cereal plots (CP) from late March (third and fourth weeks) and early April. In late April (third and fourth weeks), turtle doves started their entrance to mixed habitats of cereals and olives (CO), and then they settled in olive groves (OG) from May to June. However, few individuals were encountered in mixed habitats of cereals-olives-urban (COU) in the second week of April, the fourth week of May, and the first week of July. In contrast, urban habitats (UH) were only crossed during flight but not visited during the breeding period (Fig. 4).

The frequentation of the habitats is a consequence of the availability of food resources principally cultivated seeds in cereal plots, nesting supports (trees) in olive orchards, and water availability in rivers and dams (Table 2). These elements are capable of ensuring the success of breeding season in turtle dove.

**Table 2:** General linear models showing a summary of models influencing the occurrence of turtle doves (response variable) in studied habitat (*N* = 740 observation points). DR, Distance to the nearest road; DB, Distance to the nearest building; DW, Distance to the nearest water source; DC, Distance to nearest cereal plot; PR, Presence of predators; CP, Presence of competitors.

Occurrence of turtle doves	Type III sum of squares	df	Mean square	F	*P*-value
Corrected model	46,740.908^a^	42	1,112.879	8,050.015	0.000
Intercept	8,275.147	1	8,275.147	59,858.325	0.000
DR	0.000	1	0.000	0.000	1.000
DB	465.949	18	25.886	187.247	0.000
DW	14,190.689	4	3,547.672	25,662.110	0.000
DC	0.000	0			
PR	0.000	0			
CP	115.810	1	115.810	837.709	0.000
Error	96.357	697	0.138		
Total	80,190.000	740			
Corrected Total	46,837.265	739			

**Note:**

a Adjusted.

### Phenology of breeding turtle doves

**Figure 5:**
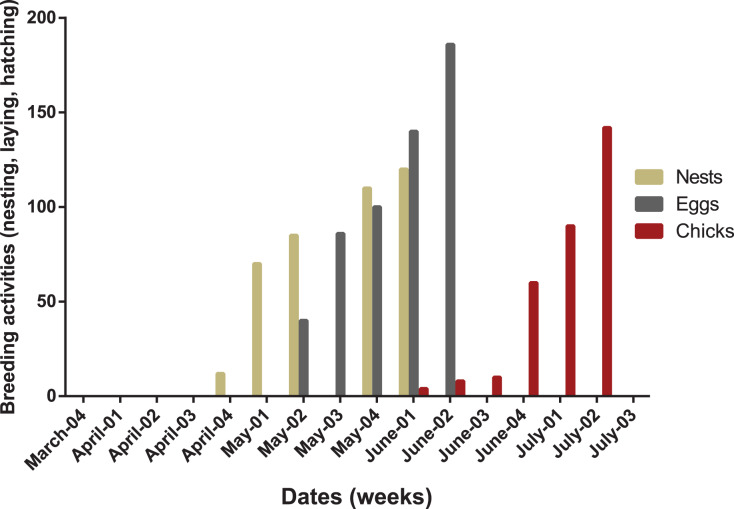
Breeding chronology of turtle doves at the Saïss plain during two seasons of 2018 and 2019.

First migratory turtle doves were witnessed during the third week of March (arrival date). They arrived as solitary individuals at first and then in groups. Breeding started approximately 3–4 weeks after arrival. The breeding period of the turtle dove in olive groves, including dates of nest construction, egg laying, and hatching is illustrated in Fig. 5. Once turtle doves had settled in the area, breeding pairs began their courtship and nesting activities. First nests were recorded during the fourth week of April. Egg laying started during the second week of April. Consequently, the first chicks fledged during the first week of May. No relationship was demonstrated between nesting, laying, and fledging dates (Table 3).

**Table 3:** General linear model relating laying and fledging to nesting dates.

Source	Sum of squares	Df	Mean square	F-ratio	*P*-value
Fledging dates	11,334.9	6	1,889.15	1.27	0.3678
Laying dates	4,770.62	1	4,770.62	3.20	0.1114
Residual	11,925.5	8	1,490.68		
Total (corrected)	28,918.4	15			

**Figure 6:**
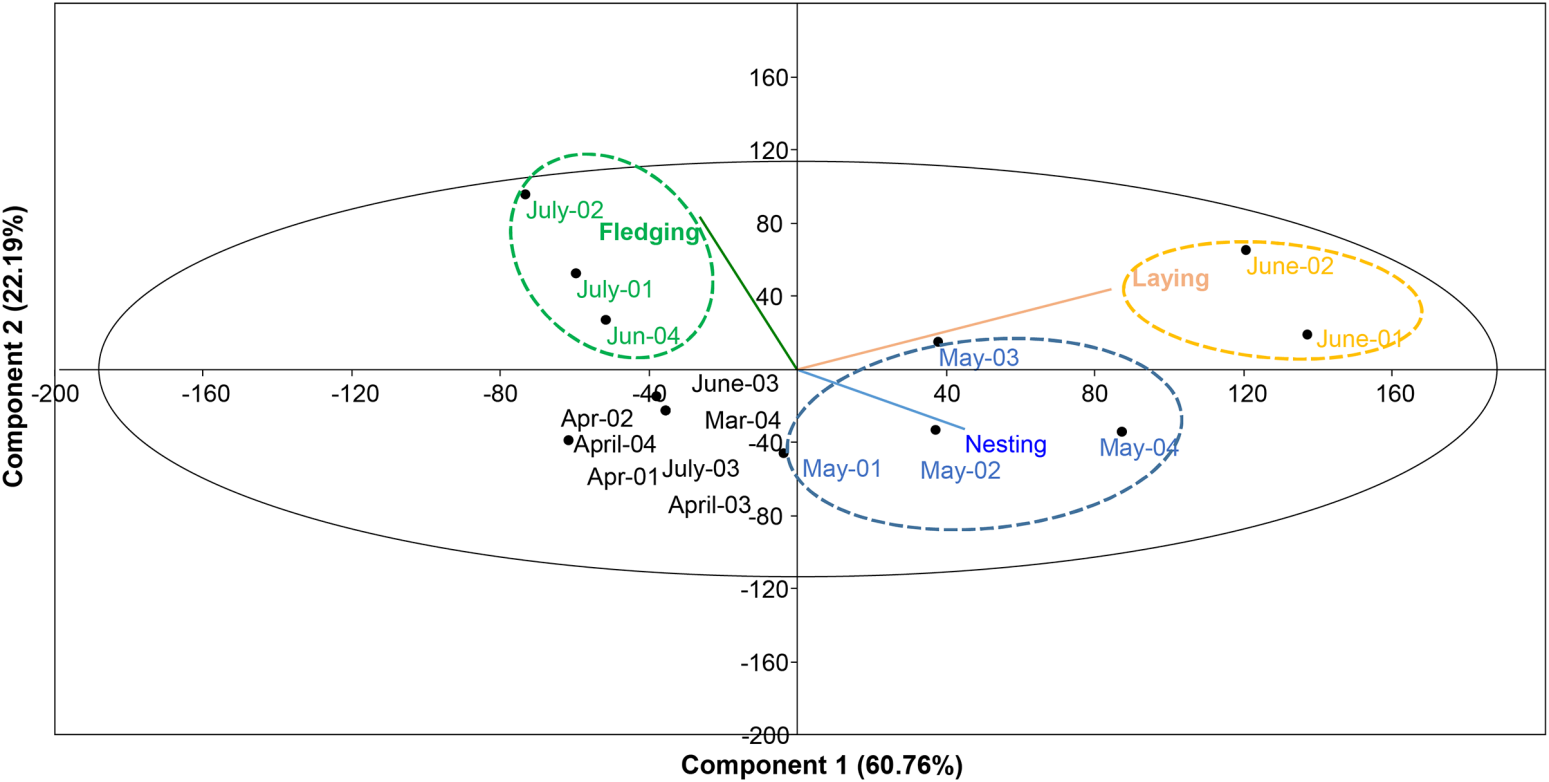
PCA plot showing the optimum breeding phases of turtle doves in study area during 2018 and 2019.

The breeding period (first nest to last chick’s emancipation) was from late April (fourth week) to second week of July. The nesting period lasted from 25 April (first nest) to 10 June (last newly built nest). The laying period lasted from 10 May (first eggs) to 15 June (last laid eggs). The fledging period started in early June and finished on 16 July. Nesting building activities occurred during four weeks of May, egg laying was observed between the first and second week of June, while fledging chicks were recorded in July (Fig. 6).

### Nest characteristics and clutch size of breeding turtle doves

**Figure 7:**
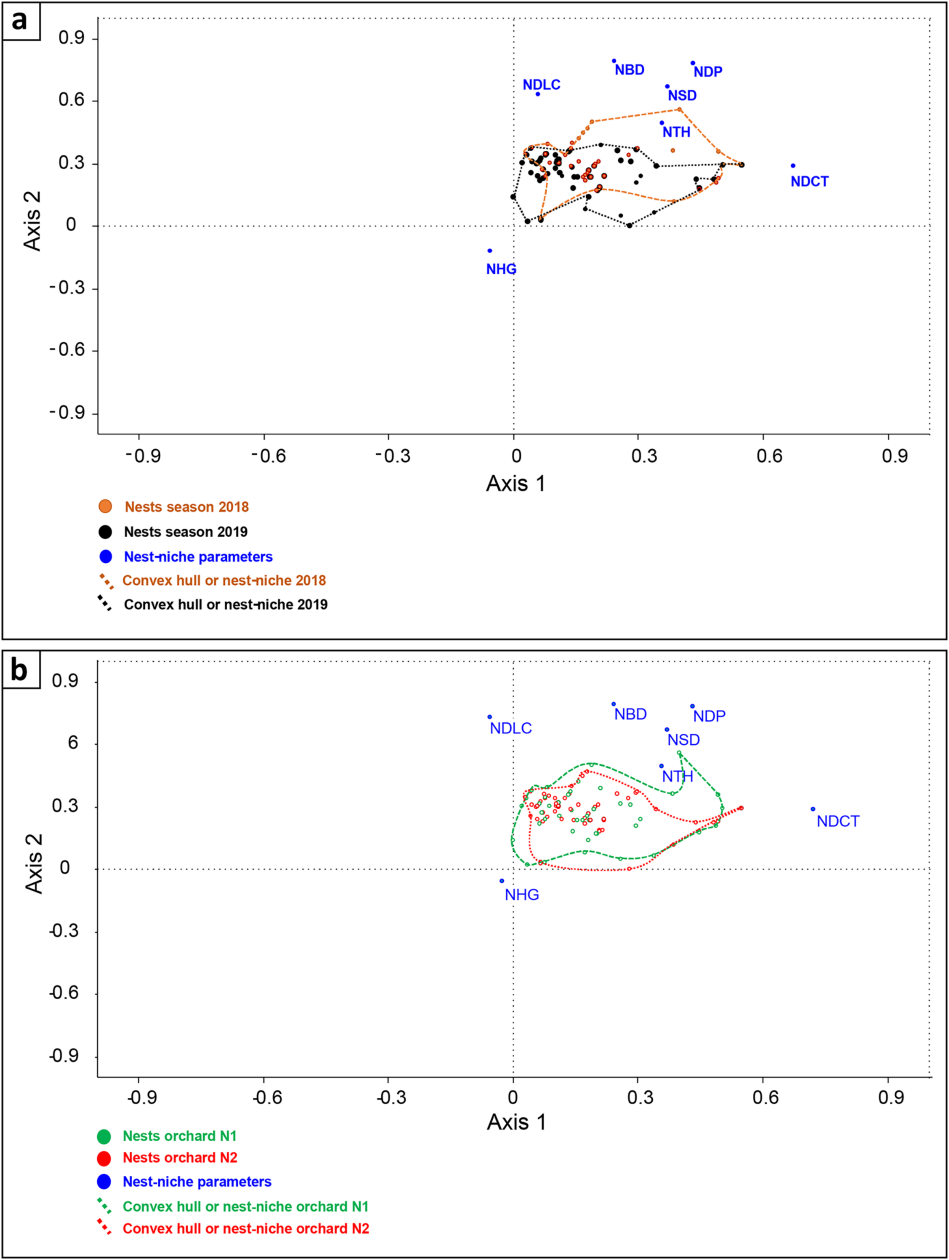
DCA Comparing Turtle dove micro-nest-niche (placement and dimension) between breeding seasons (A) (2018 and 2019) (NBD, nest big diameter; NDP, nest depth, NHG, nest height; NTH, nesting tree height; NSD, nest small diameter; and NDLC, lower canopy distance) and orchards (B) (N1 and N2).

Among recorded nests, 97 were located in central areas of olives (47 in orchard N1 and 50 in orchard N2), while only 23 were located in marginal olives (13 in orchard N1 and 10 in orchard N2). Generally, the clutch size of the turtle doves ranged between one and two eggs with a medium size of 1.98 ± 0.13 eggs (laid eggs/active nests). Dove nests had flat-shaped form, with a significant axis (external diameter) of 18.65 ± 2.60 cm (*n* = 120 nests), a minor axis (internal diameter) of 15.05 ± 2.65 cm, and a cup depth of 5.08 ± 1.53 cm. Mean nest height above the ground was 225.30 ± 48.87 cm and 106.10 ± 38.28 cm away from the central tree trunk. Only nest height above the ground (NHG) was correlated to lower canopy distance (NDLC) (Table 4). Micro-nest-niche was variable between 2018 and 2019: in 2018, nests had superior outer diameter (NBD), small diameter (NSD), nest depth (NDP), and were placed on elevated olives (NTH), while nest-niche of 2019 was characterized by taller nest placement above the ground (NHG). In contrast, nest-niche was similar between selected orchards (Fig. 7).

**Table 4:** Pearson correlation coefficients among nest placement characteristics and dimensions of turtle dove in orchards habitats. NBD, nest big diameter; ND, nest depth; NH, nest height; NTH, nesting tree height; NSD, nest small diameter; *P*, *P*-value.

	NBD	NDCT	NDLC	NDP	NHG	NSD	NTH
**NBD**		0.121	−0.624	−0.124	−0.646	0.386	−0.295
***P***		0.716	0.061	0.708	0.052	0.246	0.376
**NDCT**	0.121		0.071	−0.486	0.243	−0.346	0.475
***P***	0.716		0.830	0.144	0.464	0.298	0.153
**NDLC**	−0.624	0.071		0.391	0.664	−0.003	0.224
**P**	0.061	0.830		0.240	0.046	0.992	0.501
**NDP**	−0.124	−0.486	0.391		−0.171	0.291	−0.297
**P**	0.708	0.144	0.240		0.607	0.381	0.372
**NHG**	−0.646	0.243	0.664	−0.171		−0.055	0.312
***P***	0.052	0.464	0.046	0.607		0.868	0.347
**NSD**	0.386	−0.346	−0.003	0.291	−0.055		−0.517
***P***	0.246	0.298	0.992	0.381	0.868		0.120
**NTH**	−0.295	0.475	0.224	−0.297	0.312	−0.517	
***P***	0.376	0.153	0.501	0.372	0.347	0.120	

### Periods of egg incubation and brood-rearing

During the laying phase, the incubation of eggs (*n* = 180) lasted on the average of 14.16 ± 1.32 days. The rearing period of nestlings (*n* = 120) lasted in an average 16.54 ± 1.76 days. Both sexes incubated eggs and reared chicks. However, two breeding cycles (clutches) were distinguished at Saïss; the first cycle started from the first week of April to the second week of May, and the second between the fourth week of Mai to the second week of July.

### Reproductive success and failure factors

The breeding success of the turtle dove at the Saïss plain is detailed in Table 5. Among the 120 monitored nests, a relatively high proportion of 73.87% were successful (active nests/built nests). Among the 186 recorded eggs, 76.34% fledged. Among the 142 fledged broods, 93.66% have been emancipated. A total of 71.50% of chicks (survived chicks/laid eggs) survived during the whole season.

**Table 5:** Comparison (ANOVA) of breeding success and causes of failure between breeding stages in turtle doves in Moroccan olive orchards. Since the number of destroyed nests, dead or unhatched chicks were small, they were excluded in the ANOVA.

Parameter	Nesting (nests)	Hatching (eggs)	Fledging (brood)	F	*P*
Total	120	186	142	–	–
Successful	93	142	133	12.128	0.001
Predated	9	30	8	10.072	0.001
Deserted	11	10	0	29.267	0.001
Destructed	7	0	0	–	–
Unhatched or Died	0	4	1	–	–

Factors causing the loss of clutches varied during the breeding phase (Table 4) but in general was quite low. Predation caused the loss of 7.5% of nests, 16.12% of eggs, and 5.63% of chicks. Desertion was observed in 9.16% of nests and 5.37% of eggs. A low portion of eggs (4 eggs) did not hatch, or a few chicks (one nestling) died from unknown causes. Importantly, predation attacks were mostly directed against eggs. Desertion of broods was higher during nesting and hatching phases. In summary, breeding success was high and was apparently not strongly impacted by predation or desertion (Table 6).

**Table 6:** General linear model relating the breeding success to two predictive factors.

Source	Sum of squares	Df	Mean square	F-ratio	*P*-value
Desertion	188.255	1	188.255	2.04	0.1711
Predation attacks	101.823	1	101.823	1.10	0.3080
Residual	1,567.23	17	92.19		
Total (corrected)	1,913.54	19			

The main predators observed in the area were reptiles (especially the Montpellier snake *Malpolon monspessulanus* and the horseshoe whip snake *Hemorrhois hippocrepis*), and birds, such as the Maghreb magpie (*Pica mauritanica*), and falcons (the Common kestrel *Falco tinnunculus* and the black-winged kite *Elanus caeruleus*). Breeding activities of turtle doves coincided with farming activities in the olive orchards, including pesticide use, cutting of branches, and irrigation. These agricultural practices caused a significant disturbance and forced many turtle doves to desert their nests, and in severe cases, nests were destroyed by farmers (seven nests were destroyed in studied habitats).

## Discussion

As far as we know, this is the first detailed study concerning the size, ecology, and productivity of breeding turtle doves in Morocco and the entire Northwest Africa which are considered as breeding and stopover zones for this vulnerable migrant game species. Our main objective was to provide detailed data on the population size (numbers and documented breeding pairs of turtle doves in the area), the selection of foraging and nesting habitats, as well as the breeding performances of the Northwest African subspecies of the turtle dove in peri-urban zone of Fez. These data are important for further comparative studies and conservation activities of turtle doves in Morocco and the entire Northwest African region.

Across two breeding seasons, 3,580 turtle doves, including 240 breeding pairs, were observed in different habitats at the Saïss plain. The mean density of observed birds (total recorded turtle doves/entire study area) was nearly 22.37 individual/10 ha, which is substantially higher than that reported in Europe; for example, in Spain the density varies between two and 10 doves/10 ha (Muñoz-Cobo et al., 2001; Rocha & Quillfeldt, 2015). In contrast, the density of nests in selected breeding olive orchards was 13.48 nests/ha, which is superior to 1.5 nests/ha and 10 nests/ha recorded respectively in olives of Saïss (Mansouri et al., 2022b) and Haouz-Tadla (Hanane, 2016) respectively, but inferior to 18 nests/ha in orange trees of Haouz and Tadla (Hanane, 2016) and 26.23 nests/ha mentioned in apple orchards of Midelt (central Morocco) (Mansouri et al., 2022b). However, the density and the occurrence of turtle doves were higher in cereals and olives as compared to other types of habitats, and doves selected mainly olive groves for nesting, cereals for foraging, and aquatic ecosystems (rivers and dams) for water sources. The preference of these environments could be a consequence of the availability of food resources (cultivated seeds of cereals), nesting supports (olive trees), and water availability (in rivers and dams) as has recently been demonstrated experimentally in Europe and Northwest Africa (Moreno Zarate et al., 2020). Equally, the availability of nesting and food resources were also instrumental in Midelt orchards, which is only 180 km away from Fez (Mansouri et al., 2019, 2021a).

Equally, breeding doves changed their habitats from their arrival to the end of breeding period. Riparian areas of aquatic ecosystems (AH) were the first habitats after arrival in March, then followed by cereal plots until the late April when turtle doves moved to habitats dominated by olives. Similar results were recorded in Midelt, where turtle doves used different habitats depending on breeding *activities* (Mansouri et al., 2019). *In* our case we suggest, that after their arrival migrant doves select aquatic and cereals for forage in order to recover lost energy from their long migratory trajectories (2,500 to 3,500 km; Eraud et al., 2013; Lormee et al., 2016; Schumm et al., 2021), while in May-July breeding doves enter to olives where trees are abundant to construct their nest, and these are confirmed in paragraph below.

After their arrival in March, migrant turtle doves occupied riparian habitats. They moved into cereal plots (CP) from late March (third and fourth weeks) and early April. In late April (third and fourth weeks), turtle doves started to visit mixed habitats of cereals and olives (CO), and then they were moved on to olive groves (OG) from May to June. However, few individuals were encountered in mixed habitats of cereals-olives-urban in the second week of April, the fourth week of May, and the first week of July. In contrast, urban habitats (UH) were only crossed during flight but not occupied during all periods of breeding.

Breeding pairs arrived at Saïss during the third week of March, which is in agreement with arrival dates currently reported for turtle doves in Northwest Africa (Mansouri et al., 2020b; Mansouri et al., 2021b), while in the Northern Slope of the Mediterranean basin, first doves arrive in Spain in mid-April (Fontoura & Dias, 1996) and in France in mid-May (Eraud et al., 2013; Lormee et al., 2016). The shift in arrival dates between North Africa (*S.t. arenicola*) and Europe (*S.t. turtur*) is suggested to be governed by the close distance between wintering quarters in sub-Sahara and North Africa and migration halts of migrant doves in North African stopovers. In fact, with use of geolocators, (Lormee et al., 2016) have demonstrated that breeding doves left their wintering quarters and perform 20 to 21 days of flight and stopovers in North African grounds (Sahara Desert and North of Morocco) before arriving to breeding habitats in Europe, and this period is suggested to delay arrivals in northern slope of the Mediterranean. In contrast, North African subspecies flies directly from wintering to breeding sites. Equally, breeding (nest construction) started during the fourth week of April and continued until August (fledging of last chicks). Similar results were reported from apple orchards at Midelt (Morocco) (Mansouri et al., 2021b) and in Ziban Oases of Algeria (Absi et al., 2015). However, in other Moroccan habitats, breeding activities of turtle doves started earlier; at Tadla (230 km to Fez), Haouz (500 km to Fez), and Taroudant (450 km to Fez) the first nests were built in March (Hanane, 2016; El Hassani, Dakki & El Ghadraoui, 2018).

In addition, we found that the breeding period of turtle doves at Saïss extends from April to July, similar to the situation at Taroudant (between March and July; (El Hassani, Dakki & El Ghadraoui, 2018)), while it is short in comparison to that reported at Midelt (between April and September) (Mansouri et al., 2021b). In summary, breeding periods of turtle doves vary among the habitats and zones in Morocco depending on the latitudinal and altitudinal locations (Mansouri et al., 2021b). Incubation and rearing periods were similar to those reported previously in olives and palms in both Morocco and Algeria (Hanane, 2017; El Hassani, Dakki & El Ghadraoui, 2018). The chronology of migration and breeding, *i.e.*, from the arrival to the post-nuptial departure, differs from corresponding turtle dove populations on the northern side of the Mediterranean basin: On the Iberian Peninsula, Doves arrived and start breeding activities between May and June (Lormee et al., 2016; Marx, Korner-Nievergelt & Quillfeldt, 2016). Equally, hatching and fledging occur later between June and July.

During breeding season, turtle doves built their nests high above the ground and on taller olive trees. Generally, this breeding strategy protects the clutches mainly from terrestrial predators (Colombelli-Négrel & Kleindorfer, 2009; Guan et al., 2018). In our case, nests of turtle doves in tall trees are protected against reptiles, mainly the Western Montpellier Snake (*Malpolon monspessulanus*) observed widely in studied orchards. A similar situation can be seen in riparian habitats and agro-ecosystems counting apple and orange plantations of Midelt and Beni Mellal, where turtle doves breed high in trees as a first line of protection against voracious reptiles (Mansouri et al., 2020b; Mansouri et al., 2021b). Furthermore, in our study the nest morphology was similar to that reported in farmland and woodland ecosystems in Morocco (Mansouri et al., 2021b; Squalli et al., 2021) and Algeria (Aitouakli & Bensaci, 2021). However, the most relevant findings of our study are the delimitation of turtle dove micro-nesting-niche, which varied between seasons and was stable between nesting orchards. The factors behind seasonal variation and orchards’ similarity are not yet decrypted, therefore, more advanced investigations are needed to clarify the implicated patterns in this issue.

To our knowledge, the reproductive success documented at Saïss (chick survival of 71.5%) is the highest rate ever mentioned for turtle doves. In fact, breeding success of turtle doves ranges between 40% and 60% in Algeria (North Africa), Spain, and Britain (principally *Streptopelia turtur turtur* in Europe) (Browne & Aebischer, 2003; Aitouakli & Bensaci, 2021). The degradation of breeding habitats and the reduction of food resources during breeding seasons were the main causes of breeding failure in all previous studies on both sides of the Mediterranean basin. In Morocco, the availability of favourable nesting trees (taller olives) and the abundance of close foraging resources (cereal fields and water streams), appear to be mainly responsible for the relatively good breeding success (Mansouri et al., 2019). If similar favorable conditions could be offered for the declining European subspecies elsewhere, a recovery of this vulnerable game bird might be possible.

Predation, desertion, and destruction of nests were the major factors reducing the breeding performance of turtle doves in different Moroccan ecosystems comprising apple orchards, riparian habitats at Midelt (180 km to Fez) (Mansouri et al., 2020b; Mansouri et al., 2021b), olive and orange orchards at Beni Mellal (450 km to Fez), which is similar to our results. However, in our study, predation was higher during the incubation of the eggs, whereas nest desertion was higher during nesting and incubation phases. These findings highlight the combined impact of human disturbance and predation on the breeding success of turtle doves. We assume that the real problem is that the breeding period of turtle doves coincides with farming activities in the olive groves. Disturbed breeding pairs usually desert their nests, as reported in orange groves at Beni Mellal (Mansouri et al., 2020a) and Tadla (Hanane, 2016).

## Conclusions

In summary, this study highlights the population size and provides details on several aspects of the breeding performances of turtle doves in habitats surrounding Fez city. Turtle doves arrived in the breeding area of Saïss in late March. An important breeding population of turtle doves frequented different habitats, including orchards, cereals, rivers, and dams. Breeding activities started lately during April and finished shortly in July. Nests were placed at an elevated height of taller olive trees to protect them mainly against reptilian predators. Reproductive success rate was higher in olives due to the abundance of foraging resources and the adopted nesting strategies (elevated nests). Clutches failed mainly due to predators, nest desertion, and human disturbance. Finally, comparative investigations are required in other habitats and under different environmental conditions to confirm these unique favourable findings, mainly the higher breeding success and turtle dove density. Similarly, the positive impact of the accessibility of both food and nesting resources on breeding pairs and nestlings is needed a deep examination; for example, the link between food searching efforts and breeding success; the impact of the distance separating feeding grounds and nesting locations on the rates of reproductive success and food research sorties of breeding pairs. All these features are suggested to describe the secret behind the highest reproductive success recorded and help in the conservation actions.

## Supplemental Information

10.7717/peerj.14375/supp-1 Supplemental Information 1 Dataset of chronology of breeding, reproductivity and predicting factors. Equally, the occurrence habitat of Turtle doves in the plain of Sais and controlling factors.

## Additional Information and Declarations

### Data Availability

The following information was supplied regarding data availability: The chronology of breeding, reproductivity and predicting factors and the occurrence habitat of Turtle doves in the plain of Sais and controlling factors are available in the Supplemental Files.

## References

[ref-1] Abode J, Shams-Esfandabad B, Abdi N, Ahmadi A, Toranjzar H (2021). Breeding biology of *Streptopelia turtur* in Iran. Plant Archives.

[ref-2] Absi K, Belhamra M, Farhi Y, Halis Y (2015). A comparison of the reproduction of collared doves *Streptopelia decaocto* and turtle doves *Streptopelia turtur* in the Ziban Oases (Biskra, Algeria). Journal of Entomology and Zoology Studies.

[ref-3] Aitouakli T, Bensaci E (2021). Breeding ecology and nest-site selection of turtle doves (*Streptopelia turtur*) in three new orchard habitats. Journal of Bioresource Management.

[ref-4] Antonini Y, Martins RP (2003). The value of a tree species (*Caryocar brasiliense*) for a stingless bee Melipona *quadrifasciata quadrifasciata*. Journal of Insect Conservation.

[ref-5] Bani L, Massimino D, Bottoni L, Massa R (2006). A multiscale method for selecting indicator species and priority conservation areas: a case study for broadleaved forests in Lombardy Italy. Conservation Biology.

[ref-6] BirdLife International (2015). Streptopelia turtur.

[ref-7] BirdLife International (2019). Streptopelia turtur.

[ref-8] Boutin JM, Lutz M (2007). Management plan for Turtle Dove (Streptopelia turtur) 2007–2009. European Commission. Luxembourg. http://www.polebocage.fr/IMG/pdf/turtle_dove_2007.pdf.

[ref-9] Browne S, Aebischer N (2003). Temporal changes in the breeding ecology of European turtle doves *Streptopelia turtur* in Britain, and implications for conservation. Ibis.

[ref-10] Browne S, Aebischer N, Yfantis G, Marchant J (2004). Habitat availability and use by turtle doves *Streptopelia turtur* between 1965 and 1995: an analysis of Common Birds Census data. Bird Study.

[ref-11] Browne SJ, Aebischer NJ (2004). Temporal changes in the breeding ecology of European turtle doves *Streptopelia turtur* in Britain, and implications for conservation. Ibis.

[ref-12] Browne SJ, Aebischer NJ (2005). Studies of West Palearctic birds: turtle dove. British Birds.

[ref-13] Calladine JR, Buner F, Aebischer N (2010). Temporal variations in the singing activity and the detection of turtle doves *Streptopelia turtur*: implications for surveys. Bird Study.

[ref-14] Chedad A, Bendjoudi D, Guezoul O (2020). New data on the wintering and sedentary life of the Euroopean turtle doves *Streptopelia turtur* in the Algerian Northern Sahara. Current Trends in Natural Sciences.

[ref-15] Cherkaoui SI, Bouajaja A, Elbanak A, Said L, Hanane S (2014). The Black-necked Grebe (*Podiceps nigricollis*): an expanding species in the Middle Atlas wetlands, Morocco. Wetlands Ecology and Management.

[ref-16] Chiatante G, Porro Z, Meriggi A (2021). The importance of riparian forests and tree plantations for the occurrence of the European turtle dove *Streptopelia turtur* in an intensively cultivated agroecosystem. Bird Conservation International.

[ref-17] Colombelli-Négrel D, Kleindorfer S (2009). Nest height, nest concealment, and predator type predict nest predation in superb fairy-wrens (*Malurus cyaneus*). Ecological Research.

[ref-18] Daoui K, Fatemi ZEA, Behnassi M, Shahid SA, Mintz-Habib N (2014). Agroforestry systems in Morocco: the case of olive tree and annual crops association in Saïs Region. Science, Policy and Politics of Modern Agricultural System: Global Context to Local Dynamics of Sustainable Agriculture.

[ref-19] Dauteuil O, Moreau F, Qarqori K (2016). Structural pattern of the Saïss basin and Tabular Middle Atlas in northern Morocco: hydrological implications. Journal of African Earth Sciences.

[ref-20] De Vries EHJ, Foppen RP, Van Der Jeugd H, Jongejans E (2022). Searching for the causes of decline in the Dutch population of European turtle doves (*Streptopelia turtur*). Ibis.

[ref-21] del Hoyo J, Collar N, Christie DA, Allen R (2014). Illustrated Checklist of the Birds of the World: Non-passerines.

[ref-22] Dondini G, Vergari S (2005). Bats: bird-eaters of feather-eaters? A contribution to debate on great noctule carnivory. Hystrix-the Italian Journal of Mammalogy.

[ref-23] Dubois M (2002). Contribution à l’étude de la tourterelle des bois (Streptopelia turtur): Biologie, Zoologie, Chasse. PhD Thesis.

[ref-24] Dunn JC, Morris AJ (2012). Which features of UK farmland are important in retaining territories of the rapidly declining turtle dove *Streptopelia turtur*?. Bird Study.

[ref-25] Dunn JC, Morris AJ, Grice PV, Peach WJ (2021). Effects of seed-rich habitat provision on territory density, home range and breeding performance of European turtle doves *Streptopelia turtur*. Bird Conservation International.

[ref-26] El Hassani A, Dakki M, El Ghadraoui L (2018). Chronologie de la reproduction de la Tourterelle des bois *Streptopelia turtur arenicola* dans la région de Taroudant (Maroc). Bulletin de l’Institut Scientifique, Rabat, Section Sciences de la Vie.

[ref-27] Eraud C, Boutin JM, Riviere M, Brun J, Barbraud C, Lormée H (2009). Survival of turtle doves *Streptopelia turtur* in relation to western Africa environmental conditions. Ibis.

[ref-28] Eraud C, Rivière M, Lormée H, Fox JW, Ducamp JJ, Boutin JM (2013). Migration routes and staging areas of trans-Saharan turtle doves appraised from light-level geolocators. PLOS ONE.

[ref-29] Fontoura AP, Dias S (1996). Productivity of the turtle dove (*Streptopelia turtur*).

[ref-30] Gruychev G, Mihaylov H (2019). Breeding density of European turtle dove (*Streptopelia turtur*) on Sakar mountain (SE Bulgaria). Turkish Journal of Zoology.

[ref-31] Guan H, Wen Y, Wang P, Lv L, Xu J, Li J (2018). Seasonal increase of nest height of the Silver-throated Tit (*Aegithalos glaucogularis*): can it reduce predation risk?. Avian Research.

[ref-32] Hair JF, Anderson RE, Tatham RL, William C (1995). Multivariate data analysis with readings.

[ref-33] Hanane S (2016). Effects of location orchard type, laying period and nest position on the reproductive performance of Turtle Doves (*Streptopelia turtur*) on intensively cultivated farmland. Avian Research.

[ref-34] Hanane S (2017). The European turtle-dove *Streptopelia turtur* in Northwest Africa: a review of current knowledge and priorities for future research. Ardeola: International Journal of Ornithology Ardeola.

[ref-35] Hanane S, Yassin M (2017). Nest-niche differentiation in two sympatric columbid species from a Mediterranean Tetraclinis woodland: considerations for forest management. Acta Oecologica.

[ref-36] Hossard L, Fadlaoui A, Ricote E, Belhouchette H (2021). Assessing the resilience of farming systems on the Saïs plain. Morocco Regional Environmental Change.

[ref-37] James G, Witten D, Hastie T, Tibshirani R (2013). An introduction to statistical learning.

[ref-38] Kessabi R, Hanchane M, Guijarro JA, Krakauer NY, Addou R, Sadiki A, Belmahi M (2022). Homogenization and trends analysis of monthly precipitation series in the Fez-Meknes Region, Morocco. Climate.

[ref-39] Kouchou A, El Ghachtouli N, Duplay J, Ghazi M, Elsass F, Thoisy JC, Bellarbi M, Ijjaali M, Rais N (2020). Evaluation of the environmental and human health risk related to metallic contamination in agricultural soils in the Mediterranean semi-arid area (Saiss plain, Morocco). Environment Earth Science.

[ref-40] Lormee H, Boutin JM, Pinaud D, Bidault H, Eraud C (2016). Turtle dove *Streptopelia turtur* migration routes and wintering areas revealed using satellite telemetry. Bird Study.

[ref-41] Mansouri I, Al-Sadoon MK, Rochdi M, Paray BA, Dakki M, Elghadraoui L (2019). Diversity of feeding habitats and diet composition in the turtle doves *Streptopelia turtur* to buffer loss and modification of natural habitats during breeding season. Saudi Journal of Biological Sciences.

[ref-42] Mansouri I, Mounir M, Squalli W, Elhanafi L, Dakki M, El Ghadraoui L (2020a). Migratory dates, breeding phenology, and reproductive success of European turtle doves between lowlands and highest breeding habitats in North Africa. International Journal of Zoology.

[ref-43] Mansouri I, Ousaaid D, Squalli W, Sqalli H, El Ghadraoui L, Dakki M (2020b). The turtle dove (*Streptopelia turtur*) in Midelt plain, Morocco: nesting preferences and breeding success versus the impact of predation and agricultural practices. Journal of Animal Behaviour and Biometeorology.

[ref-44] Mansouri I, Squalli W, Achiban H, Mounir M, El Ghadraoui L, Dakki M (2022a). Segregation of breeding habitats and feeding resources among five north African game species in Midelt province, Morocco. Biologia.

[ref-45] Mansouri I, Squalli W, El Agy A, Ben Hichou B, El Hassani A, El Ghadraoui L, Dakki M (2021a). Avifauna Diversity in the Gate between Humid Atlas and Saharan Desert: Midelt Province, Morocco. International Journal of Zoology.

[ref-46] Mansouri I, Squalli W, El Agy A, El-Hassani A, El Ghadraoui L, Dakki M (2021b). Comparison of nesting features and breeding success of turtle dove *Streptopelia turtur* between Orchards and Riparian habitats. International Journal of Zoology.

[ref-47] Mansouri I, Squalli W, El Agy A, Salai KE, Bouayad K, Benhichou B, El Hassani A, El Ghadraoui L, Dakki M (2022b). Analysis of Moroccan breeding and wintering population of the vulnerable European turtle dove *Streptopelia turtur*: breeding habitats, wintering sites and governing factors. Scientific African.

[ref-48] Marx M, Korner-Nievergelt F, Quillfeldt P (2016). Analysis of ring recoveries of European turtle doves *Streptopelia turtur*—Flyways, migration timing and origin areas of hunted birds. Acta Ornithologica.

[ref-49] Moreno Zarate L, Arroyo B, Peach W (2021). Effectiveness of hunting regulations for the conservation of a globally-threatened species: the case of the European turtle-dove in Spain. Biological Conservation.

[ref-50] Moreno Zarate L, Estrada A, Peach W, Arroyo B (2020). Spatial heterogeneity in population change of the globally threatened European turtle dove in Spain: the role of environmental favourability and land use. Diversity and Distributions.

[ref-51] Muñoz-Cobo J, Moreno J, Romero C, Ruiz M (2001). Análisis cualitativo y cuantitativo de las comunidades de aves en cuatro tipos de olivares en Jaén (I) comunidades primaverales. Boletín de sanidad vegetal Plagas.

[ref-52] Prakas P, Butkauskas D, Švažas S, Bea A, Yanenko V, Ragauskas A, Vaitkuvienė D (2021). The genetic diversity and structure of the European turtle dove *Streptopelia turtur*. Animals.

[ref-53] Rocha G, Quillfeldt P (2015). Effect of supplementary food on age ratios of European turtle doves (*Streptopelia turtur* L.). Animal Biodiversity and Conservation.

[ref-54] Schumm YR, Bakaloudis D, Barboutis C, Cecere JG, Eraud C, Fischer D, Hering J, Hillerich K, Lormée H, Mader V (2021). Prevalence and genetic diversity of avian haemosporidian parasites in wild bird species of the order Columbiformes. Parasitology Research.

[ref-55] Squalli W, Mansouri I, El Hassani A, El Agy A, Assouguem A, Slimani C, Fadil F, Dakki M (2021). Macro-habitat, micro-habitat segregation and breeding success of the ‘vulnerable’ native European turtle dove and the ‘invasive’ Eurasian collared dove from a North African agricultural area. Biologia.

[ref-56] Squalli W, Mansouri I, Ousaaid D, Hmidani M, Achiban H, Fadil F, Dakki M (2022). New data on breeding strategies and reproductive success of the globally threatened turtle dove co-occurring with the “Competitive” collared dove and the “Predatory” Maghreb Magpie in Olive Orchards. International Journal of Zoology.

[ref-57] Tellería JL, Carbonell R, Fandos G, Tena E, Onrubia A, Qninba A, Aguirre JI, Hernández-Téllez I, Martín CA, Ramírez Á (2020). Distribution of the European turtle dove (*Streptopelia turtur*) at the edge of the South-Western Palaearctic: transboundary differences and conservation prospects. European Journal of Wildlife Research.

